# Clostridial C3 Toxins Target Monocytes/Macrophages and Modulate Their Functions

**DOI:** 10.3389/fimmu.2015.00339

**Published:** 2015-06-30

**Authors:** Holger Barth, Stephan Fischer, Amelie Möglich, Christina Förtsch

**Affiliations:** ^1^Institute of Pharmacology and Toxicology, University of Ulm Medical Center, Ulm, Germany; ^2^Institute of Organic Chemistry III, University of Ulm, Ulm, Germany

**Keywords:** C3, Rho, macrophage, cellular uptake, targeted drug delivery

## Abstract

The C3 enzymes from *Clostridium* (*C*.) *botulinum* (C3bot) and *Clostridium limosum* (C3lim) are single chain protein toxins of about 25 kDa that mono-ADP-ribosylate Rho-A, -B, and -C in the cytosol of mammalian cells. We discovered that both C3 proteins are selectively internalized into the cytosol of monocytes and macrophages by an endocytotic mechanism, comparable to bacterial AB-type toxins, while they are not efficiently taken up into the cytosol of other cell types including epithelial cells and fibroblasts. C3-treatment results in disturbed macrophage functions, such as migration and phagocytosis, suggesting a novel function of clostridial C3 toxins as virulence factors, which selectively interfere with these immune cells. Moreover, enzymatic inactive C3 protein serves as a transport system to selectively deliver pharmacologically active molecules into the cytosol of monocytes/macrophages without damaging these cells. This review addresses also the generation of C3-based molecular tools for experimental macrophage pharmacology and cell biology as well as the exploitation of C3 for development of novel therapeutic strategies against monocyte/macrophage-associated diseases.

## Introduction: Bacterial C3 Proteins

Various Gram-positive bacteria produce and secrete C3 proteins (~25 kDa, pI > 9) that selectively mono-ADP-ribosylate the small GTPases Rho-A, -B, and -C. C3 ADP-ribosyltransferases catalyze the covalent transfer of the ADP-ribose group from the cosubstrate NAD onto Asn-41 of Rho (1, 2). In 1987, Aktories and co-workers described the first C3 protein (C3bot), which is produced from *Clostridium botulinum* C and D strains (3, 4). It became evident that there are two isoforms, C3bot1 and C3bot2 with 60% sequence identity. Further, C3 proteins were identified in *Clostridium limosum* (C3lim) (5), *Bacillus cereus* (C3cer) (6), as well as *Staphylococcus aureus* (C3stau) (7). C3stau *alias* epithelial differentiation inhibitor (EDIN) includes three isoforms (A, B, and C), which share ~35% sequence identity to C3bot1 and ADP-ribosylates RhoE in addition to Rho-A, -B, and -C (7).

The pathophysiological role of the clostridial C3 proteins is not clear so far, but it was suggested that C3stau might play a role as virulence factor, since more C3stau-producing *S. aureus* were isolated from patients with impetigo, diabetic foot ulcers, and other skin infections than from healthy carriers (8–11). In contrast to the clostridia, *S. aureus* enters mammalian cells and releases C3stau into the host cell cytosol, where the C3stau-catalyzed ADP-ribosylation of Rho results in reorganization of the actin cytoskeleton (12). In tissues, this mode of action causes disruption of cell–cell contacts and barrier functions, which allows the dissemination of *S. aureus* through the disturbed tissue barriers into deeper tissues, as already demonstrated in animal models (12, 13). Moreover, in endothelial cells, the C3stau-mediated reorganization of the actin cytoskeleton results in the formation of large transendothelial channels, so-called macroapertures (TEMs) (14–16), which increase the endothelial permeability and facilitate the dissemination of *S. aureus* from blood to deeper tissue layers.

## C3 Enzymes Mono-ADP-Ribosylate Rho and Inhibit Rho Signaling

Up to date, C3 enzymes represent the only known specific Rho inhibitors and were widely used in biochemistry, cell biology, and experimental pharmacology as highly valuable molecular tools to investigate the role of Rho-signaling *in vitro* and in living cells (17, 18). However, it soon became evident that C3 proteins are not efficiently taken up into the cytosol of the tested cell types including epithelial cells and fibroblasts and it was suggested that C3 proteins might be exoenzymes rather than typical exotoxins, which become internalized only by non-specific mechanisms, such as pinocytosis, when cultured cells were treated for long incubation periods (>24 h) with high C3 concentrations (>10 μg/ml) in the medium (19). Therefore, C3 proteins were introduced into the cytosol of cells by artificial methods including microinjection, transfection, or by the use of molecular transporters like cell-penetrating peptides, viruses, or portions of bacterial AB-toxins (18, 20).

In line with the observation of their very limited and non-specific uptake in the tested cell types, no binding and translocation subunit was found in the C3 proteins, which could mediate their uptake into the host cell cytosol. Structure analysis of C3bot and C3stau revealed that these proteins consist of a single enzyme domain with a catalytic core containing the conserved NAD binding site and a catalytic pocket with an α-helix bent lying over two antiparallel β-sheets, which form a central cleft (21, 22) and show high structure similarity with the catalytic domains of binary actin-ADP-ribosylating toxins (18, 22, 23). Moreover, like the binary actin-ADP-ribosylating toxins, C3 enzymes contain the highly conserved amino acids, which were also identified in other ADP-ribosyltransferases: the STS-motif 174Ser-Thr-Ser176, which is flanked by Arg128 and Glu214 (22) and the “catalytic glutamate” Glu214, which plays a central role in the covalent transfer of ADP-ribose onto Asn41 of Rho. Furthermore, the binary actin ADP-ribosylating toxins and the C3 enzymes contain the so-called ADP-ribosylating toxin turn-turn (ARTT) motif, two adjacent protruding turns, which play a role for the toxin-specific recognition of actin or Rho: at position 212 in turn 2, the Rho-modifying C3 enzymes have a Gln residue, the actin-ADP-ribosylating toxins a Glu residue (22, 24). The precise molecular mechanisms of the ADP-ribosylation as well as the function of the individual amino acid residues of the ADP-ribosyltransferases is described in more detail in highly acknowledged reviews (18, 25).

Besides the Rho ADP-ribosylation, C3 acts on RalA, a member of the Ras GTPase family, in a non-enzymatic manner independent from its ADP-ribosyltransferase activity. C3 directly binds to RalA (6), which keeps RalA in its inactive GDP-bound conformation and prevents the activation of downstream RalA effectors (26, 27). However, the cellular consequences of this C3–RalA interaction are not known so far.

## Molecular and Cellular Consequences of the C3-Catalyzed Rho ADP-Ribosylation

The C3 enzymes specifically mono-ADP-ribosylate the isoforms Rho A, -B, and -C at Asn-41 and prefer Rho–GDP as substrate (28, 29) because in this structure, Asn41 is accessible to C3 (30). As a consequence, the ADP-ribosylated Rho–GDP binds more efficiently to GDI, which traps Rho in the Rho–GDI complexes in the cytosol [Ref. (31), for review on Rho regulation see Ref. (32)]. This prevents the translocation of cytosolic Rho to the cytoplasmic membrane and consequently its activation to Rho–GTP and the subsequent activation of the various cellular Rho-effector molecules (33, 34). This disturbed Rho signaling is the reason underlying most of the cellular effects observed after treatment of cells with C3. However, it should be kept in mind that due to the very limited cellular uptake of C3 proteins, all the experiments with cultured cells or tissues summarized below, were performed either by incubating the cells for a long time with high concentrations of C3 protein, or by introducing C3 into the cytosol via artificial approaches.

In 1989, Chardin and co-workers described that C3-treatment of Vero cells results in a characteristic change of the cell morphology with cell-rounding, formation of long protrusions, and reorganization of the actin filaments (35). Importantly, this was the first evidence that Rho signaling might be associated with the structure of actin filaments in mammalian cells. Due to its substrate specificity, C3 proved to be a very useful tool to unravel the role of Rho for the organization of the actin cytoskeleton and for actin-dependent processes in various eukaryotic cell types. It was found that C3-treatment inhibited endocytosis, exocytosis, cytokinesis, cell cycle progression, modulated the neuronal plasticity, and induced apoptosis (25, 36, 37). In leukocytes, the C3-catalyzed Rho-inhibition inhibited migration (38–40), adhesion (41, 42), and phagocytosis (43), suggesting a pathophysiological role of C3 proteins toward such immune cells. However, the role of C3 proteins remained unclear, mainly because of their limited cellular uptake.

The increasing knowledge on the C3-mediated effects resulted in the pharmacological exploitation of C3 for novel therapeutic strategies. The C3-catalyzed inhibition of Rho signaling has protective effects on neurons of the central nervous system because it prevented the ephrin-A5-induced growth cone collapse of such cells *in vitro* (44). Moreover, independent of its ADP-ribosyltransferase activity, C3bot – but not C3lim – exhibits neurotrophic effects on cells of the central nerve system, as demonstrated for murine hippocampal neurons (45–48), but not on cells of the peripheral nerve system (49). The recombinant cell-permeable C3bot protein BA-210 (Cethrin™), which acts as Rho inhibitor, became a drug for treatment of acute spinal cord injury in men. This Rho-inhibitor showed promising effects after local application in pre-clinical animal studies (50) and is evaluated by the U.S. National Institutes of Health in a phase IIb clinical trial for its efficacy and safety.

### Clostridial C3 toxins selectively enter monocytes/macrophages and allow for targeted pharmacological modulation of rho-dependent processes in these cells

Based on reported inhibitory effects of C3 on basic leukocyte functions, a potential pathophysiological role of C3 toward immune cells was suggested and in 2010, Barth and co-workers identified monocytes/macrophages as target cells for C3bot and C3lim (19). It was demonstrated that within short incubation periods (e.g., 3 h), comparatively low concentrations of C3bot or C3lim (e.g., 0.5–1 μg/ml) were efficiently taken up into the cytosol of cultured monocytes and macrophages, such as murine (J774A.1, RAW 264.7) and human cell lines, and primary cultured human macrophages after their differentiation from blood monocytes, while under comparable experimental conditions, no relevant uptake of clostridial C3 toxins into the cytosol of cultured epithelial cells and fibroblasts was observed. Most likely, C3bot and C3lim enter the cytosol of monocytes/macrophages by a specific endocytotic mechanism because their uptake was decreased after pretreatment of cells with bafilomycin A1 (19, 51), which inhibits endosomal acidification. Taken together, these results suggest that the clostridial C3 proteins might act on monocytes/macrophages like fully functional bacterial exotoxins but the precise molecular mechanisms underlying their cellular uptake and intracellular transport are not known so far. However, Just and co-workers, who confirmed and extended the studies on the interaction of clostridial C3 proteins with macrophages, got experimental evidence that C3 binds to proteinaceous structures on J774A.1 macrophages, which exhibit significantly more C3-binding sites compared to other cell types (52). Moreover, vimentin might be involved in the uptake of C3 protein into macrophages (52).

**Figure 1:**
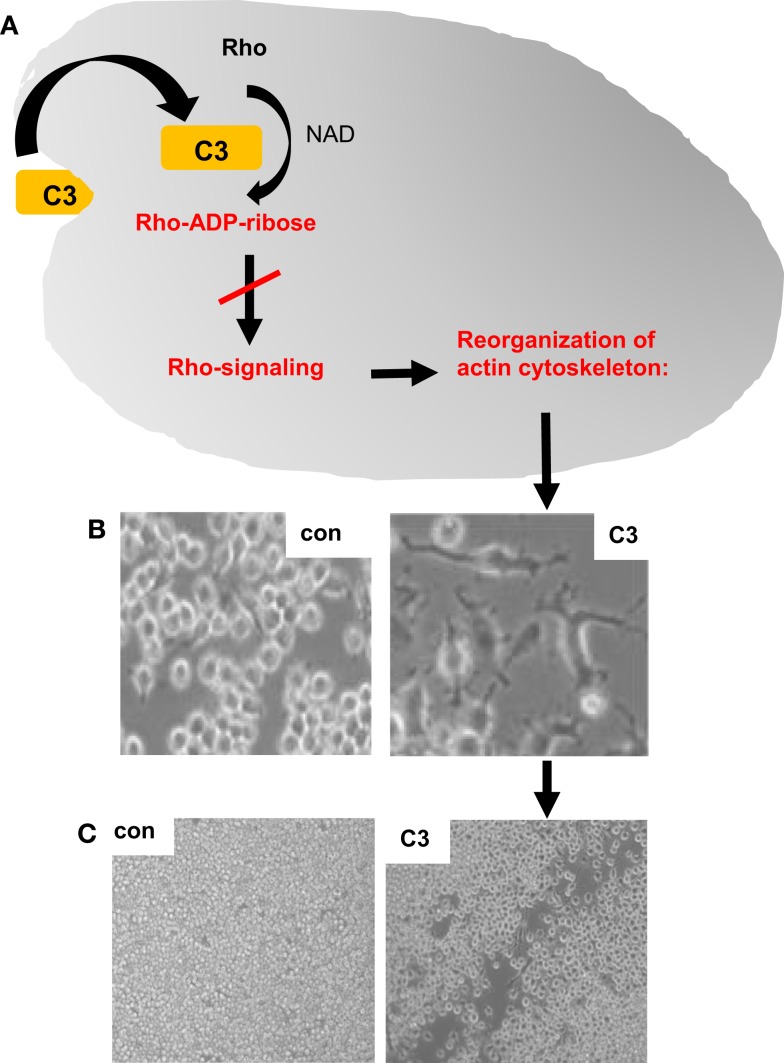
**Effects of clostridial C3 toxin on macrophages**. The uptake of clostridial C3 toxin into the cytosol of macrophages causes the C3-catalyzed ADP-ribosylation of Rho and inhibition of Rho signaling, which results in a reorganization of the actin cytoskeleton [schematically depicted in **(A)**], a dramatic change of the cell morphology **(B)** and an inhibition of the migration of cultured macrophages, as shown by the scratch test experiment **(C)**. NAD, nicotinamide adenine dinucleotide; con, untreated J774A.1 macrophages, which were grown for 24 h after the removal of a portion of the cells by a scratch; C3, J774A.1 cells treated for 24 h with 300 nM C3bot1 after removal of some cells by a scratch. After treatment with C3bot1, the scratch remains open, indicating less macrophage migration of C3-treated macrophages compared to untreated control cells.

The C3-treatment of macrophages resulted in characteristic morphological changes due to a reorganization of the actin cytoskeleton (19), as shown in Figures 1A,B. Importantly, this effect strictly depended on the C3-catalyzed ADP-ribosylation of Rho in the cytosol of the macrophages as enzymatically inactive C3botE174Q had no effect on cell morphology but was taken up into macrophages comparable to wild-type C3 (19). The C3-mediated impairment of Rho-signaling inhibits essential macrophage functions, such as phagocytosis (53) and migration [Ref. (54), Figure 1C], suggesting an immunosuppressive mode of action of the clostridial C3 proteins.

Moreover, treatment of RAW264.7 macrophages with recombinant C3bot and C3lim proteins prevented their differentiation to osteoclast-like cells and C3-treatment of already differentiated and fully active osteoclast-like cells, which can be considered as “specialized macrophages,” inhibited their resorbing activity *in vitro* (55). Interestingly, a recombinant C3lim fusion protein (C2IN–C3lim), which contains C2IN, an enzymatically inactive portion of C2I, the enzyme component of the binary actin ADP-ribosylating *C. botulinum* C2 toxin (56), was much more efficient then C3bot and C3lim regarding the targeted pharmacological manipulation of osteoclast formation and activity *in vitro* (55); most likely, this fusion protein is more efficiently internalized into the macrophages then the wild-type C3 proteins. Taken together, the recombinant C3 proteins and fusion proteins represent attractive candidates for targeted pharmacological manipulation of osteoclast formation and activity with great potential for the development of novel anti-resorptive therapies in low bone mass diseases, such as osteoporosis.

### Clostridial C3 proteins for targeted monocyte/macrophage-selective drug delivery

**Figure 2:**
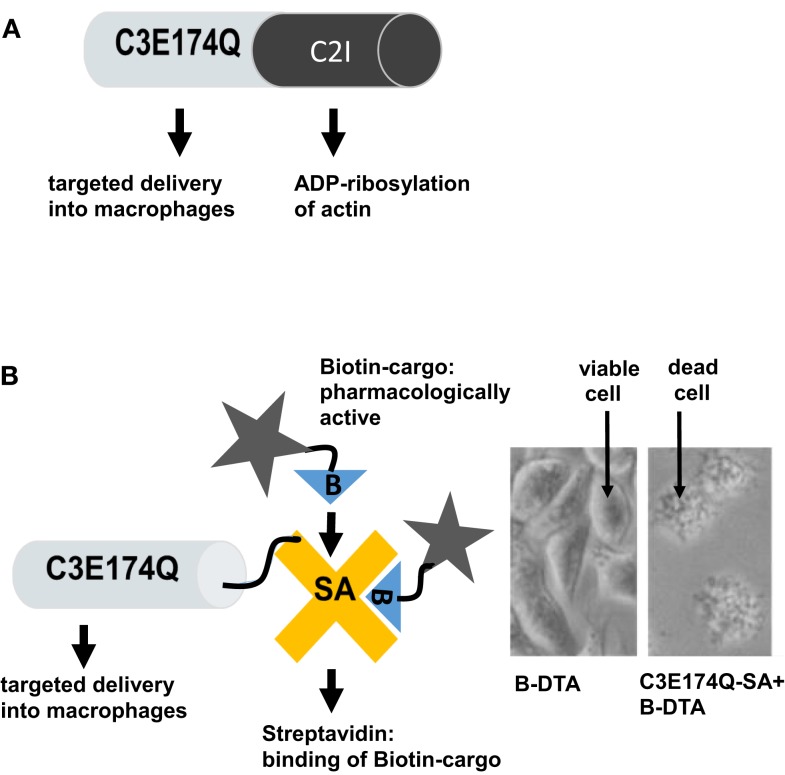
**Recombinant fusion proteins based on enzymatically inactive C3botE174Q for the targeted delivery of pharmacologically active molecules into the cytosol of macrophages and modulation of macrophage functions**. **(A)** Schematic representation of the recombinant C3botE174Q-C2I fusion toxin, which specifically ADP-ribosylates G-actin in macrophages, resulting in depolymerization of the actin filaments and inhibition of actin-dependent cell functions. **(B)** Schematic representation of the recombinant C3botE174Q-Streptavidin (indicated as C3botE174Q-SA) fusion protein, which serves as transporter for the targeted delivery of biotinylated (indicated as B-) pharmacologically active cargo molecules into the cytosol of macrophages, which are alone not taken up, in order to specifically modulate macrophage functions (left panel). Right panel: the enzyme domain of diphtheria toxin (DTA), which ADP-ribosylates elongation factor 2 in cells and triggers cell death, was biotinylated and used as a model cargo for proof-of-concept. J774A.1 macrophages incubated with biotin-labeled DTA (B-DTA) or with B-DTA, which was previously bound to the transporter molecule C3botE174Q-SA. When B-DTA was delivered into the cytosol by C3botE174Q-SA the cells died, while cells treated with B-DTA alone stayed viable [modified from Christow et al. (58)].

Due to the fact that comparatively low concentrations of the clostridial C3 proteins are much more efficiently taken up into monocytes and macrophages then in other cell types, such as epithelial cells or fibroblasts, enzymatically inactive C3 protein should represent an optimal transporter for the targeted delivery of pharmacologically active (macro)molecules including therapeutic proteins and peptides into the cytosol of monocytes/macrophages. Therefore, Barth and co-workers developed and characterized novel transport systems based on enzymatically inactive C3bot1E174Q (57), which is efficiently taken up into monocytes/macrophages (19), but does not exhibit adverse effects or induces macrophage activation (51). For proof-of-concept, a pharmacologically active enzyme, namely, the actin ADP-ribosylating *C. botulinum* C2I, was genetically fused as a reporter enzyme to C3botE174Q and the uptake of the resulting recombinant C3botE174Q-C2I (see Figure 2A) into the cytosol of cultured macrophages was investigated. Indeed, C3bot1E174Q served for delivery of C2I into the cytosol of macrophage-like cell lines and primary human macrophages derived from blood monocytes. Incubation with C3bot1E174Q-C2I in the culture medium resulted in the ADP-ribosylation of actin in the cytosol of the macrophages and consequently in the depolymerization of F-actin (51), a clear indication that enzymatically active C2I reached the cytosol. Importantly, C3bot1E174Q-C2I had no effect on epithelial cells or fibroblasts, indicating the cell-type selectivity of this fusion toxin (51). Moreover, an application of C2I alone into the medium had no effect on the macrophages because C2I is not taken up into cells. In conclusion, enzymatically inactive C3 protein was established as a novel macrophage/monocyte-selective transport system. The generated recombinant actin-inhibitor C3bot1E174Q-C2I can serve for the targeted modulation of actin-dependent processes in monocytes and macrophages, such as the inhibition of migration and phagocytosis in the context of macrophage-associated diseases.

Prompted by this first successful exploitation of C3 for protein delivery, novel modular transporters based on biotin/streptavidin technology were developed (58, 59), as depicted in Figure 2B. Here, the C3botE174Q moiety mediates the specific transport of streptavidin into the cytosol of monocytes/macrophages. Streptavidin was either coupled to C3botE174Q by chemical crosslinking (59) or by genetic fusion (58) and serves as a delivery platform for biotin-labeled cargo molecules, which are then released in the host cell cytosol to elicit their pharmacological effects, as demonstrated for the enzymatic domain DTA of diphtheria toxin (58), which triggers cell death by inactivating the elongation factor EF-2, which is essential for protein biosynthesis (60) (see Figure 2B). In conclusion, enzymatically inactive C3 protein serves as a transport system for delivery of “foreign” proteins including pharmacologically active enzymes into the cytosol of cultured monocytes/macrophages.

## Conclusion

Recombinant C3 toxins and C3 fusion toxins proved to represent valuable tools to modulate Rho- and actin-associated cell functions in monocytes and macrophages and the novel C3-based transport systems are attractive carriers for targeted drug delivery into these cells. The detailed characterization of the cellular uptake and the cellular consequences of the C3-based molecules in clinically relevant animal models, for example, after local application of C3 molecules coupled to tailored supramolecular nanocarriers (61, 62), will show whether the very promising results obtained *in vitro* and *ex vivo* can be exploited for the targeted pharmacological modulation of cellular functions in the context of monocyte/macrophage-associated diseases *in vivo*, such as inflammation, infection, or low bone mass diseases.

## Conflict of Interest Statement

The authors declare that the research was conducted in the absence of any commercial or financial relationships that could be construed as a potential conflict of interest.
